# Reducing LPS content in cockroach allergens increases pulmonary cytokine production without increasing inflammation: A randomized laboratory study

**DOI:** 10.1186/1471-2466-11-12

**Published:** 2011-02-23

**Authors:** Sudha Natarajan, Jiyoun Kim, Jacqueline Bouchard, William Cruikshank, Daniel G Remick

**Affiliations:** 1 Department of Pathology and Laboratory Medicine, Boston University School of Medicine, 670 Albany Street, 720 Harisson Avenue, Boston, MA 02118, USA; 2 The Pulmonary Center, Boston University School of Medicine, Boston, MA 02118, USA; 3 Department of Pathology, University of Michigan, 1301 Catherine Street, Ann Arbor, MI 48109, USA

## Abstract

**Background:**

Endotoxins are ubiquitously present in the environment and constitute a significant component of ambient air. These substances have been shown to modulate the allergic response, however a consensus has yet to be reached whether they attenuate or exacerbate asthmatic responses. The current investigation examined whether reducing the concentration of lipopolysaccharide (LPS) in a house dust extract (HDE) containing high concentrations of both cockroach allergens [1] and LPS would attenuate asthma-like pulmonary inflammation.

**Methods:**

Mice were sensitized with CRA and challenged with the intact HDE, containing 182 ng of LPS, or an LPS-reduced HDE containing 3 ng LPS, but an equivalent amount of CRA. Multiple parameters of asthma-like pulmonary inflammation were measured.

**Results:**

Compared to HDE challenged mice, the LPS-reduced HDE challenged mice had significantly reduced TNFα levels in the bronchoalveolar lavage fluid. Plasma levels of IgE and IgG1 were significantly reduced, however no change in CRA-specific IgE was detected. In HDE mice, plasma IgG2a levels were similar to naïve mice, while LPS-reduced HDE mice had significantly greater concentrations. Reduced levels of LPS in the HDE did not decrease eosinophil or neutrophil recruitment into the alveolar space. Equivalent inflammatory cell recruitment occurred despite having generally higher pulmonary concentrations of eotaxins and CXC chemokines in the LPS-reduced HDE group. LPS-reduced HDE challenge induced significantly higher concentrations of IFNγ, and IL-5 and IL-13 in the BAL fluid, but did not decrease airways hyperresponsiveness or airway resistance to methacholine challenge. *Conclusion: *These data show that reduction of LPS levels in the HDE does not significantly protect against the severity of asthma-like pulmonary inflammation.

## Background

Asthma is a chronic allergic disorder characterized by IgE production, airway eosinophilia and bronchial hyperresponsiveness[2]. Numerous studies have shown that allergens are not solely responsible for the severity of the asthmatic response (reviewed in[3]). Microbial components, such as lipopolysaccharide (LPS) from Gram negative bacteria, are ubiquitously present in the environment, including the ambient air. In addition to being potent activators of innate immunity, these compounds have been shown to modulate asthma severity[4]. The presence of microbial components often is associated with environmental and household cleanliness, however the hygiene hypothesis postulates that the lack of exposure to these pathogens at a young age increases susceptibility to allergen sensitization, accounting for the dramatic increases in allergic diseases in the developed world [5,6].

Several studies have been carried out investigating the role LPS plays in allergen sensitization. These studies suggest that in ovalbumin (OVA) models, increasing doses of LPS protect against eosinophilia and AHR[7,8]. Recently, more evidence has emerged implicating reduced tumor necrosis factor-α (TNFα) production as the mechanism responsible for this protection. Eisenbarth. et. al. showed that OVA sensitization in toll-like receptor-4 (TLR4) deficient mice did not result in IgE production or eosinophil recruitment, however these responses could be restored by exogenous administration of TNFα shortly after sensitization[8]. Additionally, mice deficient in TNF-receptor associated factor-1 do not develop eosinophilia or AHR in response to OVA sensitization and challenge[9].

While LPS plays a key role in allergen sensitization, the synergistic effect of LPS and allergen during the effector phase of asthma remains unclear. We sought to determine whether the removal of LPS from house dust obtained from the home environment can protect against the severity of murine airways-inflammation. We employed a clinically relevant model of asthma-like pulmonary inflammation based on immunization with a CRA[1] extract containing LPS, followed by challenge with a house dust extract (HDE) collected from the home of an asthmatic child. This HDE contains high levels of both CRA and LPS, and can induce the cardinal features of asthma-like inflammation, such as airway eosinophilia, airways hyperresponsiveness and IgE production[10,11]. In order to investigate the role of LPS at the time of allergen challenge, LPS was removed from the HDE using a commercially available column. The current study shows that reduction of the LPS content in the HDE exacerbates cytokine production, reduces IgE, but does not alter AHR, airway eosinophilia or neutrophil recruitment.

## Methods

### Animals

Female BALB/c mice 9-12 weeks old were purchased from Jackson Laboratories (Bar Harbor, ME) and maintained under standard laboratory conditions. The mice were housed in a temperature and humidity controlled room with 12 hour light/dark cycles. Food and water were allowed *ad libitum*. All experiments were performed according to the National Institutes of Health guidelines and were approved by the Boston University and University of Michigan Institutional Animal Care and Use Committees.

### Reduction of LPS content in House Dust Extract

LPS was removed from the house dust extract (HDE) using the EndoTrap Blue column (Profos AG, Regensburg, Germany), by modifying the manufacturer's protocol. HDE was diluted 1:1 in sterile PBS. The depletion column was equilibrated with the provided equilibration buffer modified to contain 400 mM NaCl. The HDE mixture was applied to the column and eluted using the provided elution buffer. The eluted mixture was immediately aliquoted and stored at -70°C until use. Concentrations of Bla g (*Blatella germanica*) 1 and Bla g2 and LPS were measured after application to the column, and appropriate dilutions prepared for *in vivo *administration.

### LPS Assay

LPS in the house dust extract and cockroach allergen preparations was assayed in pyrogen free water using the endpoint *Limulus *amoebocyte lysate (LAL) assay (Lonza, Basel Switzerland, 50-647 U). 96-well microplates and substrate solutions were warmed to 37°C. 50 μL of sample and standard were added to the plate in duplicate followed by 50 μL LAL regent. The plate was incubated at 37°C for 10 min. 100 μL of substrate solution was then added to each well and the plate was incubated at 37°C for 6 minutes. The reaction was stopped with 50 μL 25% glacial acetic acid and the absorbance was read at 405 nm.

### Asthma induction protocol

House dust collection and processing was performed as previously described[11]. We used a commercial CRA preparation for immunizations since there are limited amounts of HDE available. Additionally, our lab has demonstrated that asthma-like inflammation induced by the HDE is CRA specific[12] and CRA administration will produce asthma-like pulmonary inflammation[13,14]. For these reasons, the commercial German cockroach-allergen[1] (Greer Laboratories, LeNoir, NC, Item # B46) was used for immunization[12]. The CRA (61.9 ng) was diluted to a final volume of 50 μL in sterile phosphate buffered saline (PBS) immediately prior to use. This mixture was emulsified in 50 μL TiterMax Gold adjuvant (CytRx, Norcross, GA). Each mouse was injected i.p. with 100 μL of the adjuvant/allergen mixture on day 0. On day 14 and day 21, mice were challenged by direct intratracheal installation of 50 μL of the intact HDE or LPS-reduced HDE, each containing 61.9 ng CRA [15]. Briefly, mice were lightly anesthetized and suspended by their front incisors on a vertical board. Their tails were taped down to support the body weight. The tongue was gently extended and the liquid was placed at the base of the oropharynx so that it was inhaled.

### Timepoints for data collection

Animals were euthanized by cervical dislocation following ketamine/xylazine anesthesia at 0, 2 or 24 hours post final allergen challenge. At the time of sacrifice, plasma, bronchoalveolar lavage, and lung homogenates were prepared. The 2 and 24 hour timepoints represent the early phase and late phase of the asthmatic response based on prior publications[11]. The 0 hour timepoint refers to animals sacrificed on day 21 without receiving the second pulmonary challenge. This timepoint was included to determine if sensitization and the first challenge caused any lasting inflammatory reaction in either group. Airway hyperresponsiveness [10] was measured 4 hours post final challenge, based on our observations of robust AHR induction at this timepoint (data not shown). These mice were then sacrificed at 24 hours.

### Airways Hyperresponsiveness and Airway Resistance

Airways changes were measured either with direct invasive techniques (Flexivent, Scireq Scientific Respiratory Equipment, Montreal, Canada) or using unrestrained whole body plethysmography (Buxco Systems, Troy, NY). For whole body plethysmography, mice were placed in the instrument chamber and allowed to acclimate for at least 5 minutes. Baseline measurements were recorded for 5 minutes. Mice were then challenged for 2 minutes with aerosolized PBS and increasing doses of methacholine (Sigma, St. Louis, MO). Each aerosol challenge was followed by 5 minutes of monitoring and data collection. The partial pressure difference between the experimental and reference chambers represented the PenH parameter, and the data presented as the percent increase above baseline PenH measurements.

These data were further verified using invasive pulmonary function tests. For measurement of mouse airway resistance, mice were anesthetized with an i.p. injection of 1:5 diluted pentobarbital (Nembutal^®^, 0.016 ml/g body weight, Ovation Pharmaceutical, Deerfield, IL). The paralytic was pancuronium (Sigma-Aldrich, St. Louis, MO) at 0.5 micrograms per gram body weight. Once adequate surgical sedation was established, determined by a firm squeeze of the foot pad, a tracheotomy was performed by insertion of an 18 g polyethylene cannula into the distal trachea. The mouse was then placed on the FlexiVent mechanical ventilator (Scireq Scientific Respiratory Equipment, Montreal, Canada) and ventilated at 190 breaths per minute with positive-end expiratory pressure set at 3 cmH_2_O. Measurement of airway resistance in response to increasing concentrations of aerosolized methacholine was obtained through periodic computer-generated "snapshot 150" forced-maneuver interruptions in ventilation. Data are then presented as resistance change from baseline (cmH_2_O per milliliter per second).

### Bronchoalveolar lavage and lung homogenate preparation

Mice were exanguinated and BAL performed by cannulating the trachea. The lung was lavaged with 2, 1 mL aliquots of warm Hank's Buffered Salt Solution (HBSS, Gibco, Grand Island, NY). Both aliquots were centrifuged and the supernatant of the first wash removed and frozen at -20°C for cytokine analysis. The supernatant from the second aliquot was discarded and the cell pellets were resuspended and combined. Total cell counts were obtained using a Beckman-Coulter particle counter model ZF (Coulter Electronics Inc., Hialeah, FL). Cytospin preparations were stained with Diff-Quick and 300 cell differential counts were performed to determine the absolute numbers of inflammatory cells. The right lung was removed, placed in ice cold protease inhibitor cocktail (Roche, Indianapolis, IN) containing 0.00005% Triton X-100 in PBS, and homogenized with 3, 10 second passes in a Brinkmann Polytron PT3000 homogenizer. An aliquot was removed and sonicated in hexadecyltrimethylammonium bromide (HTAB) buffer for myeloperoxidase assay. A separate aliquot was removed and sonicated in 0.5% cetyltrimethylammoniumchloride (CTAC) (Sigma, St. Louis, MO) for eosinophil-specific peroxidase assay. The homogenized and sonicated mixtures were centrifuged at 15,000 g for 15 min. The homogenate supernatant was removed and stored at -20°C for cytokine analysis and the supernatant from the sonicated fractions was used immediately for peroxidase assays.

### Myeloperoxidase and Eosinophil Peroxidase Assays

Myeloperoxidase (MPO) and eosinophil peroxidase (EPO) assays were performed as described previously[16], with some modifications. EPO was performed by diluting the supernatant of the mixture sonicated in CTAC 1:3 in 10 mM HEPES, pH 8, in quadruplicate in a 96-well plate. 150 μL ice cold stop solution (4N H_2_SO_4 _+ 2 mM resorcinol) was added to 2 of the sample wells. 75 μL of substrate solution containing 6 mM KBr, 1.5 mM *o*-phenylenediamine (Sigma, St. Louis, MO, P9029), and 0.3% H_2_O_2 _in 50 mM HEPES, pH8, was added to the two remaining sample wells and incubated in the dark for 30 seconds. 150 μL ice cold stop solution was added and the absorbance read at 490 nm.

MPO was measured by diluting the supernatant of the mixture sonicated in HTAB 1:5 in 10 mM citrate buffer, pH 5 in quadruplicate. 150 μL ice cold stop solution (4N H_2_SO_4_) was added to 2 of the sample wells. 75 μL substrate solution containing 0.3 mM 3,3',5,5'-Tetramethylbenzidine, 120 mM resorcinol (an eosinophil peroxidase inhibitor), and 0.007% H_2_O_2 _in ddH_2_O was added to the two remaining sample wells and incubated in the dark for 2 minutes. 150 μL ice cold stop solution was added and the absorbance read at 450 nm. Data is expressed as ΔOD reflecting the difference in absorbance between the average of the sample and background wells.

### ELISA

Cytokines and chemokines were measured by ELISA as previously described[17]. Matched antibody pairs and recombinant standards were purchased from R&D Systems (Minneapolis, MN). Lung homogenate samples were assayed with the addition of 20% normal lung homogenate to the standards to adjust for the increased background caused by non-specific matrix effects. Antibody ELISA reagents (IgG and IgE) were purchased from Bethyl Laboratories (Montgomery, TX) and assays were performed by the same standard protocols as for cytokines and chemokines. Cockroach allergen ELISAs were performed as previously described[12] with antibody pairs and recombinant standards from Indoor Biotechnologies (Charlottesville, VA).

CRA-specific IgE was measured by coating ELISA plates with CRA (Greer Labs) over-night at 4°C. Plates were washed and non-specific binding blocked for 2.5 hours at room temperature on an orbital shaker. Plates were washed an incubated with 1:10 dilutions of plasma overnight at 4°C. Plates were washed and incubated with HRP-goat anti mouse IgE for 1 hour at room temperature on an orbital shaker. Plates were developed using 3,3',5,5'-Tetramethylbenzidine as substrate and read as previously described[12]. Results are represented as ΔOD (OD_465 _- OD_590_). Since CRA-specific IgE standards are not currently available, plasma from a naïve mouse was run in parallel in each assay for baseline.

### Cysteinyl-Leukotriene Immunoassay

Cysteinyl leukotrienes in the BAL fluid were measured by an Enzyme-linked Immunoassay Kit (Cayman Chemicals, Ann Arbor, MI) according to the manufacturer instructions. All samples were run at two dilutions. %B/B_0 _values in the linear range of the standard curve were accepted. Sample values that did not fall in this range were appropriately diluted and rerun.

### Statistical Analysis

All data are represented as mean ± SEM. Statistical significance was determined by unpaired Student's *t*-test or One-way ANOVA with Turkey's post test using GraphPad Prism version 4.0.3. (GraphPad Software, San Diego, CA). Statistical significance was achieved when *p *< 0.05.

## Results

### Depletion of LPS from the house dust extract

Dust was collected from the homes of asthmatic children as described previously[11]. This dust was shown to contain significantly higher concentrations of cockroach allergens Bla g1 and Bla g2 in comparison to other indoor and outdoor allergens[12]. This extract, when used in previous studies at a 1:10 dilution (containing a total of 61.9 ng CRA) successfully induced the hallmark features of asthma-like inflammation, including airways hyperresponsiveness, eosinophilia and plasma IgE[18-20]. In the present study, we specifically sought to determine whether removal of the potent innate immune activating agent LPS from the HDE at the time of allergen challenge would attenuate the severity of pulmonary inflammation in sensitized mice. To address this question, LPS in the HDE was reduced as described in materials and methods. Unfortunately, our previous attempts at LPS removal using polymyxin B columns (Pierce, Rockford, IL) also removed 99.9% of Bla g1 from the HDE, and these preparations were not appropriate for the present studies (Table 1). Although use of the EndoTrap column in this study slightly reduced CRA concentrations, through appropriate dilution, we were able to maintain the same total amount of 61.9 ng CRA for all treatments (Table 2).

**Table 1:** Total amounts of CRA and LPS in the intact HDE and in the HDE treated with Polymyxin B or EndoTrap to deplete LPS.

	Bla g1 (ng/ml)	Bla g2 (ng/ml)	LPS (ng/ml)
Intact HDE	4,938	11,710	18.2

After Polymyxin B Column	1.4	6,400	1.0

After Endotrap Column	2,058	6,113	1.7

**Table 2:** Amounts of CRA and LPS present in both the immunization and challenge mixtures.

Procedure	Reagent	Total CRA (ng/dose)	LPS (ng/dose)
Immunization	Commercial CRA	61.9	11.6

Challenge	LPS-reduced HDE	61.9	3.0

Challenge	HDE	61.9	182.1

### TNFα production in BAL fluid post final challenge

**Figure 1:**
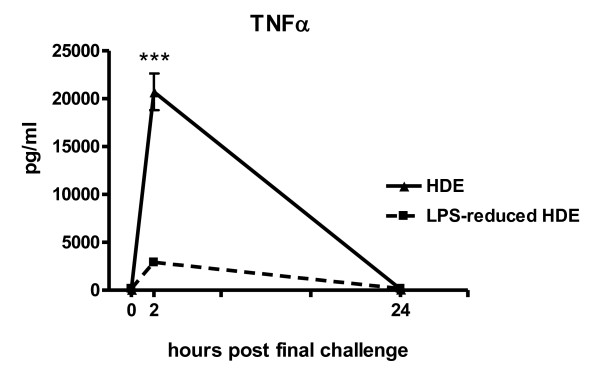
**TNFα production in bronchoalveolar lavage fluid**. TNFα was measured by ELISA at the timepoints indicated. The 0 h sample was collected prior to the second pulmonary challenge. Data are represented as the mean ± SEM, in some data points the symbol is larger than the SEM. Experiments were done a minimum of 3 times with 3-4 mice per group, per experiment. ***p ≤ 0.0001 comparing the LPS-reduced HDE group to the HDE group by Student's *t*-test.

Sensitization to cockroach allergens plays a key role in the development of childhood asthma[21], and the level of LPS present in the home has also been shown to have a major effect on the severity of respiratory diseases[5,10,22]. Asthma-like inflammation was induced by i.p. immunization with 61.9 ng CRA, containing 11.6 ng of LPS. Mice were challenged on days 14 and 21 with either the intact HDE (182.1 ng of LPS) or LPS-reduced HDE (3.0 ng of LPS). Although different allergen mixtures are used for sensitization and challenge, we have previously shown that HDE immunization specifically induces an allergic response to CRA[12]. Consistent with the low levels of LPS present in the LPS-reduced HDE, TNFα production was significantly decreased in this group at 2 hours post final challenge (Figure 1). We have previously shown that this early timepoint reflects the point at which TNFα production peaks in the BAL fluid[19].

### Role of LPS in antibody production

**Figure 2:**
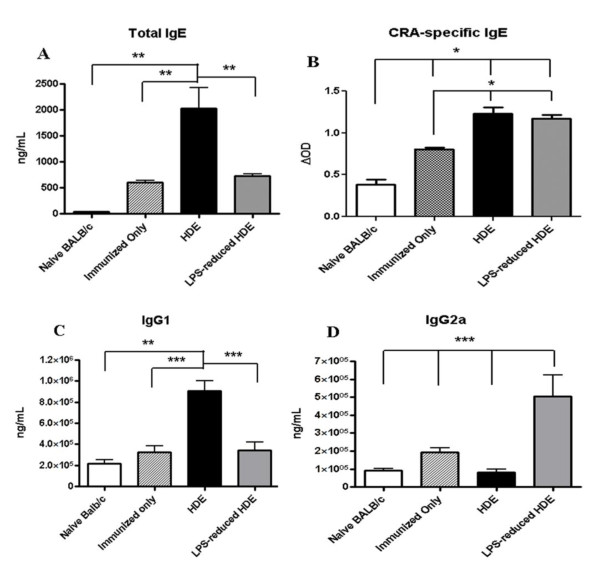
**Plasma antibody levels in immunized and challenged mice**. Plasma levels of A) IgE, B) CRA-specific IgE, C) IgG1, and D) IgG2a were measured by standard ELISA from EDTA-plasma collected 24 hours post final challenge (day 22). The immunized-only group was sensitized on Day 0 and plasma was collected on day 22. Data are represented as the mean ± SEM. Statistical significance was assessed by one-way ANOVA followed by Turkey's post test as indicated in each panel. Experiments were done a minimum of 3 times with 3-4 mice per group, per experiment. ** *p *< 0.001.

The presence or absence of TNFα has been shown to affect the antibody response to allergen[8]. We sought to determine whether the reduced levels of TNFα produced in response to challenge with the LPS-reduced HDE had an effect on antibody production in this model. Intact HDE challenge resulted in robust IgE production while challenge with LPS-reduced HDE did not induce IgE production beyond that seen in mice receiving only the CRA immunization (Figure 2A). Despite the global increase in IgE, measurement of CRA-specific IgE showed no difference between LPS-reduced HDE challenge and HDE challenge (Figure 2B). IgG1 production was similar to total IgE production with reduced levels in the mice challenged with the LPS-reduced HDE compared to immunized-only mice (Figure 2C). These changes in immunoglobulins were not found with all classes, since LPS-reduced HDE challenge induced significantly higher levels of plasma IgG2a compared to the immunized-only group (Figure 2D).

### Inflammatory cell recruitment in the BAL fluid post allergen challenge

**Figure 3:**
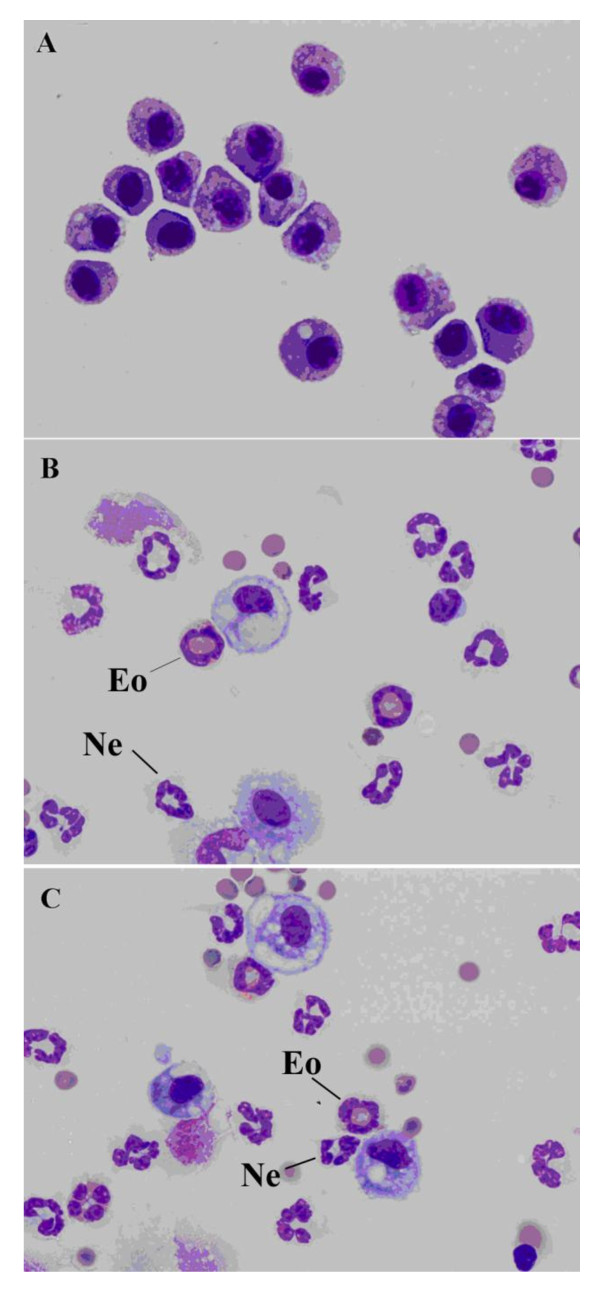
**Cytospin preparations of cells recovered from BAL fluid**. A) Naïve mice, B) HDE challenged mice and C) LPS-reduced HDE challenged mice at 24 hours post final challenge. Each is represented at 1000×. Representative eosinophils (Eo) and neutrophils (Ne) are indicated in the figure.

**Figure 4:**
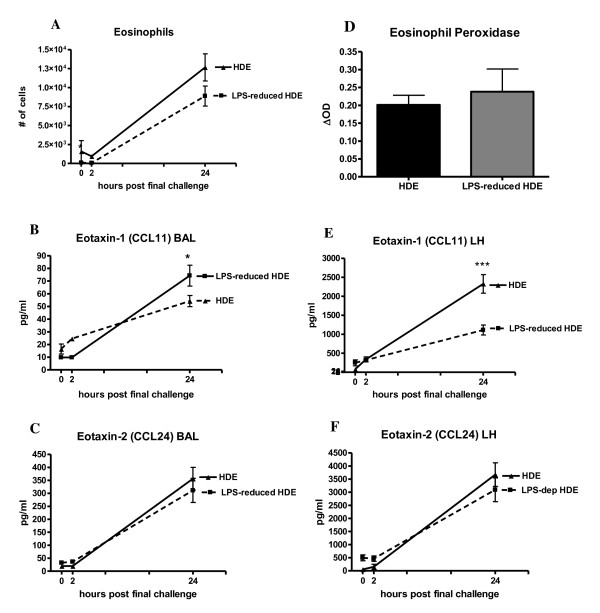
**Eosinophil recruitment and production of eosinophil-specific chemokines in response to allergen challenge**. Cytospin preparations from cells collected in the BAL fluid at the timepoints indicated were made and 300 cell differential counts performed to determine the absolute numbers of eosinophils (A). Concentrations of the chemokines B) CCL11 and C) CCL24 in the BAL fluid were measured by ELISA. D) Eosinophil peroxidase activity was measured in the lung tissue after BAL at 24 hours post final challenge. Concentrations of E) CCL11 and F) CCL24 in the lung homogenate were assayed by ELISA. Data are represented as the mean ± SEM. Experiments were done a minimum of 3 times with 3-4 mice per group, per experiment, in some data points the symbol is larger than the SEM. * *p *< 0.05 and *** *p *≤ 0.0001 HDE vs. LPS-reduced HDE by Student's *t*-test.

The composition of inflammatory cells in the BAL fluid was assessed at various timepoints after the final intratracheal challenge. Cytospin preparations demonstrate distinct inflammatory cell morphology. Eosinophils were characterized by a ring-shaped nucleus with pink cytoplasmic amyloid staining, while neutrophils show a lobular nucleus with no cytoplasmic staining. Similar numbers of eosinophils and neutrophils in the mice challenged with the intact HDE or the LPS-reduced HDE, in contrast to that of naïve mice containing predominantly macrophages (Figure 3). We observed a slight reduction in eosinophil numbers in the BAL fluid of the LPS-reduced HDE group, but this change did not reach statistical significance (Figure 4A). In the BAL fluid, the eosinophil-specific chemoattractant eotaxin-1 (CCL11) was significantly increased at 24 hours post final challenge in the LPS-reduced HDE mice, however no difference in eotaxin-2 (CCL24) was observed (Figure 4). We also assayed eosinophil-specific peroxidase activity (EPO) in the lungs of mice (after BAL) as a measure of eosinophils within the lung interstitium. No significant difference in EPO was measured between the two groups (Figure 4D). A statistically significant decrease in eotaxin-1 (CCL11) was measured in the lung homogenate of the LPS-reduced HDE group (Figure 4E), but no difference was measured in eotaxin-2 (CCL24) between groups (Figure 4F). While statistically significant differences in eotaxin-1 are observed in both the BAL and lung homogenate, it should be noted that eotaxin-2 is produced at much higher concentrations in both the alveolar compartment and the lung tissue, rendering an equivalent biological outcome (eosinophil recruitment) in both groups.

**Figure 5:**
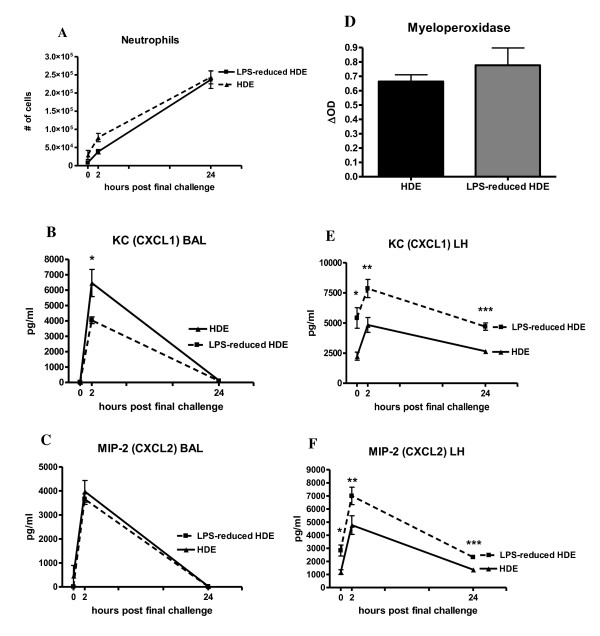
**Neutrophil recruitment and production of neutrophil-specific chemokines in response to allergen challenge**. Cytospin preparations from cells collected in the BAL fluid at 24 hours post final challenge were made and 300 cell differential counts performed to determine the absolute numbers of A) neutrophils. Concentrations of chemokines B) CXCL1 and C) CXCL2 in the BAL fluid were measured by ELISA. D) Myeloperoxidase activity was measured in the lung tissue after BAL at 24 hours post final challenge. Concentrations of E) CXCL1 and F) CXCL2 in the lung homogenate were assayed by standard ELISA. Data are represented as the mean ± SEM, in some data points the symbol is larger than the SEM. Experiments were done a minimum of 3 times with 3-4 mice per group, per experiment. * *p *< 0.05, ** *p *≤ 0.001 and *** *p *≤ 0.0001 comparing the LPS-reduced HDE group to HDE the group by Student's *t*-test.

We also assayed the number of neutrophils recruited in response to allergen challenge. Interestingly, comparable numbers of neutrophils were present in both groups despite reduced levels of LPS in the LPS-reduced HDE (Figure 5A). This suggests that other components of the HDE are able to activate the innate immune response in order to recruit neutrophils. The concentrations of the neutrophil chemoattractants CXCL1 and CXCL2 were assayed as the mechanism of neutrophil recruitment into the alveolar space and lung tissue. BAL levels of CXCL1 are significantly decreased at 2 hours in mice challenged with the LPS-reduced HDE, however no difference was seen in CXCL2 (Figure 5). Despite this slight decrease in chemokine production, we postulate that the concentrations at which they are being produced is adequate to recruit equivalent numbers of neutrophils shown in Figure 5A.

Myeloperoxidase activity (MPO) in the lung tissue was not changed between groups. (Figure 5D). Significant increases were measured in both CXCL1 and CXCL2 in the lung homogenate in the LPS-depleted HDE at all timepoints assayed (Figure 5E). This again suggests that although the chemokines were lower in the intact HDE, the concentrations were sufficient to sequester neutrophils within the lung interstitium. No differences in macrophage or lymphocyte numbers were seen in the BAL and no difference was seen in neutrophil numbers at 2 h post final challenge (data not shown).

### Induction of airways hyperresponsiveness after allergen challenge

**Figure 6:**
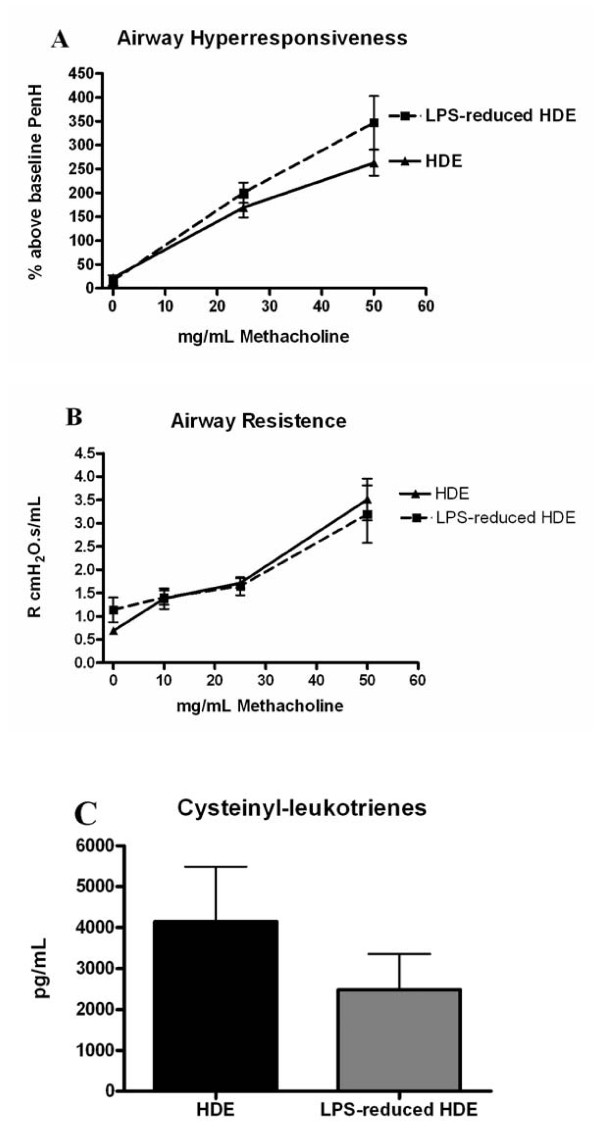
**Airways hyperresponsiveness, airway resistance and cysteinyl leukotrienes post allergen challenge**. A) For non-invasive measurement of AHR, mice were challenged with increasing concentrations of aerosolized methacholine at 4 hours after the final challenge and PenH values are represented as percent above baseline. B) For invasive measurements, mice were challenged with methacholine and pulmonary resistance is expressed as R cmH_2_O.s/mL. C) Cysteinyl leukotriene concentrations were measured in the BAL fluid 2 hours post final challenge. Data are represented as the mean ± SEM, in some data points the symbol is larger than the SEM. Experiments were done a minimum of 3 times with 3-4 mice per group, per experiment.

Several studies have shown that both LPS inhalation and pulmonary TNFα administration induce AHR, however little data exist regarding the synergistic role of allergen and endotoxin on AHR[23-25]. Therefore, we sought to determine whether the levels of LPS in the HDE would affect AHR severity. Challenge with increasing concentrations of aerosolized methacholine 4 hours post final challenge did not result in any difference in AHR between groups (Figure 6A). The 4 hour timepoint was assessed based on observations in our laboratory showing robust AHR induction at this timepoint. No increase in AHR was measured comparing normal mice and sensitized-only mice (data not shown). Given the controversy associated with non-invasive whole body plethysmography for assessing AHR, we confirmed our findings using invasive lung function tests. As shown in Figure 6B, invasive tests demonstrated no significant difference in airway resistance between groups at all methacholine concentrations tested. We also assessed BAL cysteinyl leukotriene (Cys-LT) concentrations at 2 hours as a mechanism for AHR, and found no significant change in BAL Cys-LT in mice challenged with LPS-reduced HDE compared to HDE challenge (Figure 6C).

### Th1 and Th2 cytokine production in the BAL post allergen challenge

**Figure 7:**
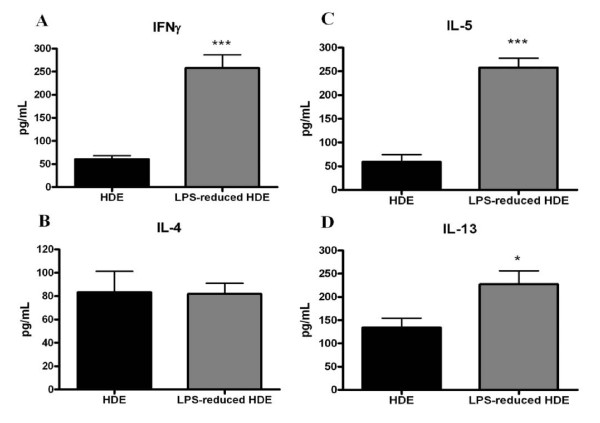
**Th1 and Th2 cytokine production in the BAL fluid at 24 hours post final challenge**. Cytokines were assayed by standard ELISA. Data are represented as the mean ± SEM. Experiments were done a minimum of 3 times with 3-4 mice per group, per experiment. * *p *< 0.05 and *** *p *≤ 0.0001 comparing the LPS-reduced HDE to HDE the group by Student's *t*-test.

It has been suggested previously that the presence of TNFα can induce increased levels of Th2 cytokines in the BAL fluid of allergen challenged mice[19]. Further, asthma is largely characterized as a Th2 mediated disease, accompanied by high IL-4 and IL-5 production and low IFNγ[26]. To determine whether the reduction in TNFα seen in LPS-reduced HDE challenged mice affected cytokine production, we assayed both Th1 and Th2 cytokines 24 hours post final challenge. In conflict with the Th2 paradigm, IFNγ gamma was significantly increased in the LPS-reduced HDE mice (Figure 7A). Surprisingly, IL-5 and IL-13 were significantly increased in the BAL from LPS-reduced HDE challenged mice although there was no difference in IL-4 levels (Figure 7B, C). IL-17 has also been implicated as a key cytokine in the asthmatic response, and is also modulated by LPS levels [27,28]. However, we measured no significant differences in IL-17 levels between groups in the BAL fluid (data not shown).

## Discussion

The effect of exposure to household dust on the development of atopy and asthma has recently become a focus of investigation; however this avenue of study has resulted in more controversy than consensus. Epidemiological evidence shows that the presence of cockroach allergens in inner city homes is a strong risk factor for the development of atopic disease [29,30]. Cockroach allergen sensitization was found to be responsible for exacerbations in a large proportion of asthmatic children in inner city Washington, D.C [21]. Further, asthma exacerbations were not the result of sensitization to other allergens such as cat dander and dust mite allergens, which were also present in these homes [1,21]. Exposure to innate immune activators such as TLR ligands, as well as environmental pollutants such as diesel particulates can also drive the adaptive immune response toward tolerance or sensitization [10,31]. This area of study is gaining significant interest in light of the increasing incidence of atopy and allergy in the developed world.

This study sought to examine whether the presence of LPS in the household environment modifies the allergic response after sensitization to cockroach allergens has been established. It is possible that our method of LPS reduction also removed other allergens from the HDE mixture which may contribute to the allergic response. While this is a real concern, it is a shortcoming of all column purification methods. We are confident that our methods resulted in physiologically relevant data given that the LPS-reduced HDE was still able to induce a robust adaptive immune response.

Recent studies have employed house dust extracts to determine their roles in asthma severity, and have suggested that these extracts can induce allergic phenotypes or can skew towards tolerance, depending upon the dose and timing of the exposure [31,32]. However, these studies focused on the immunomodulatory effects of the house dust extracts on mice presensitized to ovalbumin, and thus study house dust as an exacerbating factor, rather than a causative agent. In contrast, the current study directly examines the synergistic effects of CRA and LPS contained in the HDE in mice presensitized to CRA, which is the major allergen in the HDE.

LPS contamination in allergen preparations has been raised as an issue in many models of allergy, however we believe that approaching these studies using household components with naturally occurring LPS more closely and accurately reflects the environment in which one develops asthma. It is not appropriate to refer to the HDE as "contaminated" with LPS, similar to how one would not say it is contaminated with cockroach allergens. In an OVA study, Watanabe et.al. demonstrated that LPS contamination of commercially available OVA preparations significantly attenuated cellular influx, AHR and IgE production [33]. The differences observed between these models might be explained by the intrinsic protease activity of the cockroach allergen Bla g2, which may enhance allergen sensitization by increasing epithelial permeability, and allergen exposure to dendritic cells [34,35]. This highlights the need to study the effect of LPS in the development of asthma-like inflammation using a relevant allergen mixture.

In the lung, TNFα is primarily produced by alveolar macrophages and secreted within 1 hour of LPS challenge [36,37]. Our results show that mice challenged with the LPS-reduced HDE produce significantly less TNFα compared to those challenged with the HDE, verifying that removal of LPS from the HDE has the expected immunological effect. Several studies have implicated TNFα and TNFα signaling in the development of adaptive immunity, particularly in the context of class switch to IgE production [8,9]. Therefore, we would expect that we did not see differences in CRA-specific IgE, given that our study focused on the effect of LPS at challenge only. In contrast, the studies by Eisenbarth et. al. and Watanabe et.al. focused on LPS at the time of sensitization, which likely has more effect on the production of antigen-specific antibodies [8,33]. This was also demonstrated in a rat model where aerosol exposure to LPS before or shortly after sensitization reduced the development of OVA-specific IgE [22]. Levels of CRA-specific IgG1 and IgG2a are of significant interest, however reliable assays for these antibodies are not currently available.

The increase in total IgE and IgG1 in the HDE-challenged mice represents an increase in polyclonal, antigen non-specific IgE. LPS alone has been shown to induce expression of activation-induced cytidine deaminase, which is required for activation of downstream signals responsible for class switch recombination to both IgG1 and IgE in B cells [38]. Other models have shown polyclonal IgE to have a role in mast cell survival and cytokine release [39].

Our data demonstrate that airways hyperresponsiveness was not diminished in the LPS-reduced HDE challenged groups despite significantly attenuated TNFα. We postulate that AHR is governed by other mediators and is not directly induced by TNFα. The role of IgE was explored in other studies that demonstrated that AHR may be modified independent of IgE alterations. Hamelmann et. al. showed robust AHR induction in both mast cell and B cell deficient mice and it has been shown that mast cell activation is not required for the induction of AHR[40,41]. Further, methacholine acts directly upon muscarinic receptors in order to induce smooth muscle contraction. We have recently shown that pulmonary exposure to high concentrations of LPS can induce modest differences in muscarinic receptor expression[42]. Therefore, it is possible that the concentrations of LPS used in this study were not sufficient to alter muscarinic receptor expression, and therefore did not alter AHR.

The role of LPS in driving allergic responses toward a Th2 phenotype has been demonstrated in several models and our data show this to be true in the context of total antibody production. However, our data show increases in both Th1 and Th2 cytokines in the LPS-reduced HDE group. One possible explanation for this is that the source of these cytokines is not only the T cells present in the lung. Other cell types such as eosinophils, epithelial cells and mast cells are contributing to the pool of cytokines present in the BAL fluid [43]. Therefore, without assessing the direct cellular source of these cytokines, it is not possible to conclude whether LPS-induced Th2 cell differentiation is impaired in this model.

Interestingly, challenge with intact HDE results in decreased BAL cytokine production. We postulate that this is modulation of the innate immune response through induction of microbial cross-tolerance. We and others have shown that cross-tolerance to TLR ligands can be induced in the lung and in pulmonary macrophages [44,45]. It is likely that simultaneous exposure to various TLR ligands present in the HDE can induce cross-tolerance and therefore attenuate the cytokine response. Removal of the potent TLR4 agonist LPS, may reduce the tolerogenic response, driving increased cytokine production.

Limited epidemiological data exist investigating whether removal of house dust can limit future asthma exacerbations. One such study carried out in the UK found no correlation between removal of house dust and amelioration of asthma symptoms in presensitized individuals. However, interventions to reduce dust in homes of children who were highly susceptible to developing atopy was effective in reducing asthma diagnosis [46,47]. Taken together our data show that in a clinically relevant model of house dust induced asthma-like inflammation, the removal of only LPS at challenge does not protect against the development of the inflammatory response. Further studies will be needed to clarify whether LPS in the home environment exacerbates asthma in presensitized individuals.

## Conclusions

Decreasing the endotoxin content endogenously present in cockroach allergens results in a complicated pulmonary inflammatory response. While there was differential regulation of cytokine, chemokines and plasma levels of immunoglobulins, there was no real change in AHR or pulmonary inflammatory cell recruitment.

## Competing interests

The authors declare that they have no competing interests.

## Authors' contributions

SN designed and executed the experiments and wrote the manuscript. JB and JK assisted with conducting the experiments, interpreting the data and reviewing the manuscript. WC assisted with the Flexivent measurements and reviewed the manuscript. DR assumed overall responsibility for the experimental design and manuscript preparation. All authors read and approved the final manuscript.

## Pre-publication history

The pre-publication history for this paper can be accessed here:

http://www.biomedcentral.com/1471-2466/11/12/prepub
